# Protein Kinase C Alpha Cellular Distribution, Activity, and Proximity with Lamin A/C in Striated Muscle Laminopathies

**DOI:** 10.3390/cells9112388

**Published:** 2020-10-31

**Authors:** Hannah A. Nicolas, Anne T. Bertrand, Sarah Labib, Musfira Mohamed-Uvaize, Pierrette M. Bolongo, Wen Yu Wu, Zofia T. Bilińska, Gisèle Bonne, Marie-Andrée Akimenko, Frédérique Tesson

**Affiliations:** 1 Department of Biology, Faculty of Science, University of Ottawa, Ottawa, ON K1N 6N5, Canada; hnico019@uottawa.ca (H.A.N.); wenyu.wu@mail.utoronto.ca (W.Y.W.); makimen@uottawa.ca (M.-A.A.); 2 Sorbonne Université, Inserm, Centre de Recherche en Myologie, UMRS 974, G.H. Pitié-Salpêtrière, 75013 Paris, France; a.bertrand@institut-myologie.org (A.T.B.); g.bonne@institut-myologie.org (G.B.); 3 Interdisciplinary School of Health Sciences, Faculty of Health Sciences, University of Ottawa, Ottawa, ON K1N 6N5, Canada; sarah.labib@canada.ca (S.L.); mmoha054@uottawa.ca (M.M.-U.); pbolongo@uottawa.ca (P.M.B.); 4 Unit for Screening Studies in Inherited Cardiovascular Diseases, National Institute of Cardiology, 04-628 Warsaw, Poland; zbilinska@ikard.pl

**Keywords:** lamin A/C, protein kinase C alpha, striated muscle laminopathies, DCM, EDMD, L-CMD

## Abstract

Striated muscle laminopathies are cardiac and skeletal muscle conditions caused by mutations in the lamin A/C gene (*LMNA*). *LMNA* codes for the A-type lamins, which are nuclear intermediate filaments that maintain the nuclear structure and nuclear processes such as gene expression. Protein kinase C alpha (PKC-α) interacts with lamin A/C and with several lamin A/C partners involved in striated muscle laminopathies. To determine PKC-α’s involvement in muscular laminopathies, PKC-α’s localization, activation, and interactions with the A-type lamins were examined in various cell types expressing pathogenic lamin A/C mutations. The results showed aberrant nuclear PKC-α cellular distribution in mutant cells compared to WT. PKC-α activation (phos-PKC-α) was decreased or unchanged in the studied cells expressing *LMNA* mutations, and the activation of its downstream targets, ERK 1/2, paralleled PKC-α activation alteration. Furthermore, the phos-PKC-α-lamin A/C proximity was altered. Overall, the data showed that PKC-α localization, activation, and proximity with lamin A/C were affected by certain pathogenic *LMNA* mutations, suggesting PKC-α involvement in striated muscle laminopathies.

## 1. Introduction

Lamins A and C are A-type lamins coded by the lamin A/C (*LMNA*) gene. They are nuclear intermediate filaments important for structural support and maintenance of various nuclear processes, such as gene expression [1]. Mutations in *LMNA* cause a group of diseases called laminopathies; 79% affect the heart and/or skeletal muscles [2]. One of the cardiac diseases that can be caused by lamin A/C mutations is dilated cardiomyopathy (DCM) with or without conduction defects (CD). DCM is defined by the enlargement of left or both ventricles and systolic dysfunction not caused by coronary artery disease or abnormal loading conditions [3]. Patients with DCM caused by lamin A/C mutations are afflicted with a more severe phenotype and have poorer prognosis compared to patients with DCM caused by mutations in other genes [4,5,6,7]. Moreover, individuals with DCM caused by *LMNA* mutations frequently present conduction defects, and/or develop heart failure requiring heart transplant [4,5,6,7,8]. Emery-Dreifuss muscular dystrophy (EDMD) and *LMNA*-related congenital muscular dystrophy (L-CMD) are skeletal muscle conditions also caused by lamin A/C mutations [9,10,11]. Both dystrophies have childhood onset and overlapping clinical presentations [12]. However, L-CMD is more severe than EDMD [12]. The cardiac phenotype (arrhythmia, cardiac dysfunction) of the L-CMD patients are also more progressive and manifests at a younger age compared to EDMD [12,13].

Previous work suggests that disruption of lamin A/C interaction with its binding partners contributes to the pathophysiology of laminopathies [14,15,16,17,18,19]. The lamin A/C binding partner protein kinase C alpha (PKC-α) is a classical member of the PKC family of serine/threonine kinases encoded by the *PRKCA* gene [20,21]. PKC-α is mostly localized in the cytoplasm, but it can translocate to various subcellular locations, including the nucleus [20,22,23]. It is presumed that PKC-α found in other subcellular locations aside from the cytoplasm is active because it is in these locations where PKC-α acts on its targets [20,22,24]. PKC-α is phosphorylated at Thr497, Thr638 and Ser657 for activation; phosphorylation of Ser657 is important for full phosphorylation, activity, and accumulation of mature PKC-α [25,26,27,28,29]. PKC-α is the major classical PKC isoform in the mouse, the rabbit and the adult human heart, and is a fundamental regulator of cardiac contraction via intracellular calcium regulation [30,31,32,33]. Change in PKC-α expression is associated with DCM or heart failure in various patient and animal model studies [34,35,36,37,38]. Genetic abolition of *Prkca* or pharmacological inhibition of PKC-α/β results in either improved cardiac function or cardio-protection from heart failure in mice, rat and pig heart failure models, thereby implicating PKC-α in cardiac function and heart failure development and progression [30,31,39,40]. PKC-α is also the major classical PKC isoform in adult mouse skeletal muscles [41]. In vitro work demonstrates its influences on myocyte structure and dynamics by regulating protein filamin C (FLNc) [42]. FLNc binds actin and is important in Z-disc assembly [42]. Phosphorylation of FLNc by PKC-α inhibits FLNc cleavage and affects FLNc dynamics and mobility; thus, regulating FLNc function [42]. Altogether, these data suggest PKC-α involvement in myocyte structure and contraction.

Aside from the A-type lamins, PKC-α also interacts with/regulates other lamin A/C partners, including certain MAP kinases [43,44,45,46,47,48]. Cardiac and/or skeletal muscle tissue samples from the EDMD *Lmna* H222P mouse model and in patients with DCM caused by lamin A/C mutations, show that members of the MAP kinase (MAPK) signaling pathway, particularly ERK 1/2 and several of its downstream targets, are highly expressed/active [49,50,51]. ERK 1/2 inhibitor treatment of symptomatic EDMD H222P mice rescues cardiac and skeletal muscle function, improves the cardiac and skeletal muscle histology and normalizes the abnormal gene expression profile; inhibitor treatment of asymptomatic H222P mice delays disease onset and lessens the severity of the disease [51,52,53,54]. Furthermore, *Erk 1* knockout alleviates the cardiac symptoms and improves survival of the H222P mice [55]. However, this improvement in cardiac function is abolished by 20 weeks of age, as ERK 2 activation gets elevated [55]. Treatment of the *Erk 1* null H222P mice with a MEK inhibitor which prevents ERK activation restored the benefits of *Erk 1* knockout, further confirming the role of ERK in the development of striated muscle laminopathies [55].

Previous studies suggest PKC-α regulation of ERK 1/2 activation [45,46,56,57]. Similarly, other members of the MAPK pathway—JNK and p38—which are also implicated in striated muscle laminopathies, are regulated by PKC-α [44,47,48]. Thus, with the central role of PKC-α in cardiac function, its interaction with A-type lamins and its regulation of several proteins associated with striated muscle laminopathies, we sought to determine the involvement of PKC-α in cardiac and muscular laminopathies. In this study, we examined PKC-α cellular distribution, activation and lamin A/C proximity in the presence of pathogenic lamin A/C mutations using cardiac and skeletal myoblast cell lines; striated muscle laminopathy mouse model myoblasts; and fibroblasts and myoblasts from two L-CMD patients carrying distinct mutations. We show that in transfected cells expressing a DCM-causing *LMNA* mutation and in skeletal myoblasts from two individuals harboring different L-CMD mutations, PKC-α was found to have increased nuclear localization compared to cells expressing WT *LMNA*. On the other hand, transfected cells expressing a truncated lamin A/C variant that lacked the PKC-α binding domain presented with PKC-α that was mostly cytoplasmic. These data show that PKC-α cellular localization is disturbed in the presence of *LMNA* mutations. Furthermore, PKC-α and ERK 1/2 activations were decreased or unchanged in patient cells and in skeletal myoblasts from two different mouse models of striated muscle laminopathies. In addition, PKC-α-lamin A/C proximity was also altered in the patient cells and mouse model myoblasts. Altogether, the results demonstrate for the first time the involvement of PKC-α in cardiac and muscular laminopathies.

## 2. Materials and Methods

### 2.1. Approved Research Protocols for Human Cells and Mice Model Cells

Collection and handling of human cells were performed in accordance with the French legislation on ethical rules. Primary mouse myoblasts were obtained following protocols conformed to French laws and were approved by the French Ministry of Higher Education and Research (approval number 00972.03)

### 2.2. Cell Culture and Maintenance

The H9C2 rat cardiac myoblasts (ATCC^®^ CRL-1446™; VA, USA), the C2C12 mouse myoblasts (ATCC^®^ CRL-1772™) and the human skin fibroblasts were cultured in complete medium made up of Dulbecco’s modified eagle’s medium (DMEM) supplemented with 10% fetal bovine serum (XE “Fetal Bovine Serum”) (FBS), 1% L-glutamine and 1% Pen/Strep at 37 °C in 5% CO_2_. The p.delK32 fibroblasts were collected as described in [58] while the p.R249W fibroblasts were collected from the patient described in [59] during surgical intervention to reduce the patient’s contractures.

Myoblasts from the homozygous H222P [60] and homozygous ΔK32 [61] mouse models were taken from the hind limb and cultured on 10% Matrigel coated plates in DMEM supplemented with 20% FBS, 10% horse serum, 0.5% chicken embryo extract and 1% penicillin/streptomycin at 37 °C in 5% CO_2_.

Human myoblasts from paraspinal (control), gluteus (p.delK32 patient) and deltoid (p.R249W patient) muscles [59] were cultured in DMEM supplemented with 16% medium 199, 20% FBS, 0.05% insulin (10 mg/mL stock), 0.025% fetuin (25 μg/mL stock), 0.005% hEGF (5 ng/mL stock), 0.005% bFGF (0.5 ng/mL stock), 0.2% dexamethasone (0.1 mg/mL stock) and 0.1% gentamicin at 37 °C in 5% CO_2_.

### 2.3. Cloning

To generate the plasmids for transfection, full length human lamins A and C cDNAs were cloned in-frame into eCFP-C1 and eYFP-C1 vectors (Takara Bio USA, Inc formerly Clontech Laboratories, Mountain View, CA, USA) respectively, as described in [62,63]. Full length human lamin A cDNA was also inserted into the eYFP-C1 vector between the BamHI and XbaI restriction sites. Electro-competent *Escherichia coli* were transformed and grown on kanamycin plates to isolate bacterial colonies expressing the recombinant plasmid. Isolated bacterial clones were further grown in LB medium with kanamycin to propagate the recombinant plasmid. Recombinant plasmid extraction was performed using the Qiagen Plasmid Maxi Kit (Qiagen, Toronto, ON, Canada) following the manufacturer’s protocol. The fidelity of the inserts was confirmed by sequencing.

### 2.4. Cell Transfection

Cells were double transfected with WT or mutant human lamins A and C using Metafectene^®^ Pro (Biontex München, Germany) following the manufacturer’s guidelines. Transfection of both human lamins A and C was performed to maintain the stoichiometric amounts of A-type lamins normally found in cells. Briefly, fresh complete medium was added to cells prior to transfection. Two tubes containing DMEM were prepared per sample. DNA was added in one of the two tubes, and Metafectene^®^ Pro was added in the other tube. The DNA solution was then carefully added to the metafectene solution. The DNA/metafectene mix was left to incubate without disturbance at room temperature (RT) for at least 20 min prior to addition to the cells. Cells were incubated in the transfection solution for 21 h.

### 2.5. Immunofluorescence

H9C2 and C2C12 cells were grown on coverslips and double transfected with WT or mutant human lamins A fused to eCFP and C fused to eYFP. At 21 h post transfection, cells were fixed with 4% paraformaldehyde (XE “paraformaldehyde”) (PFA) for 20 min at RT and washed in PBS three times (5 min/wash). Following fixation, cells were blocked and permeabilized with 0.1% TritonX100 in 5% FBS for 20 min at RT. Cells were then incubated with the primary antibody for 1.5 h at 37 °C and then washed in PBS three times (5 min/wash). Fluorescently-labeled secondary antibody incubation was performed for 1 h at 37 °C in the dark. The cells were then washed in PBS three times (5 min/wash). Coverslips were mounted on slides using ProLong™ Gold Antifade Mountant (Life Technologies Thermo Fisher Scientific Canada). Double immunostaining was performed in the same manner with the samples being incubated with two primary antibodies first, followed by incubation with two secondary antibodies. Supplementary Table S1 lists the antibodies that were used.

Human and mouse model cells were fixed in 4% PFA for 10 min and washed in 1X PBS. Samples were then blocked and permeabilized with 5% BSA-PBST for 30 min at RT. Primary antibody incubation was for 1.5 h at RT and secondary antibody incubation was for 1 h at RT in the dark. All washes were done three times (5 min/wash) in PBST except the one after fixing, which was done in PBS. Coverslips were mounted on slides using ProLong™ Gold Antifade Mountant. Supplementary Table S1 lists the antibodies that were used.

### 2.6. Confocal Microscopy

Z-stack images of transfected H9C2 and C2C12 cells were taken using the Zeiss LSM510 AxioImager M1 confocal microscope using the Plan Apochromat 63× (1.40 NA) oil immersion objective. The following lasers were used: 458 nm (emission filter 510–565 nm), 514 nm (emission filter 520–555 nm) and 633 nm (emission filter 650LP).

Z-stack images of the human and mouse model cells were taken using the Nikon A1RsiMP Confocal Workstation using the Plan Apochromat 60× (1.40 NA) oil immersion objective. The following lasers were used: 405, 488, 561 and 640 nm. When applicable, channel series was activated to prevent spectral bleed through between the channels.

ImageJ 1.47v 2.0.0-rc-43/1.52n (for Windows 64-bit) was used to analyze the raw fluorescent cell images. PKC-α fluorescence intensity was measured in two regions in the cytoplasm and one region in the nucleus. Background signal was measured from three different non-cellular regions on the image. The area of the selected region was kept constant throughout the measurements and between groups. PKC-α fluorescence intensity measurements were performed on one of the middle z-stacks to ensure that the selected nuclear region is solely nuclear. Adjusted PKC-α fluorescence intensity was determined by taking the integrated density of the selected region − (area of the selected region × mean fluorescence of background readings) [64]. The adjusted nuclear and cytoplasmic PKC-α fluorescence intensities were then used to calculate the net PKC-α fluorescence intensity, which is the difference between the adjusted nuclear and cytoplasmic PKC-α fluorescence intensities (i.e., net nuclear PKC-α fluorescence intensity = adjusted nuclear PKC-α fluorescence intensity − mean of the adjusted cytoplasmic PKC-α fluorescence intensities). A positive value signifies an increase in nuclear PKC-α localization and a negative value signifies that PKC-α is mainly cytoplasmic. Adobe Photoshop CC 2019 was used to convert single channel images to black and white, increase image brightness for ease of visualization, annotate the images and prepare the figures.

### 2.7. Cell Sorting

Transfected cells expressing both human eCFP-lamin A and eYFP-lamin C were washed in PBS prior to addition of trypsin to detach the cells from the plate. Cells were then suspended in DMEM only and filtered through a 40 μm mesh (to minimize cell clumps). Transfected cells were then sorted and isolated using a Beckman Coulter MoFlo XDP (Ottawa Hospital Research Institute Flow Cytometry and Cell Sorting Facility, Ottawa, ON, Canada).

### 2.8. Protein Extraction and Western Blot

Nuclear protein extraction of double transfected and sorted C2C12 cells was performed using the Active Motif nuclear extraction kit (Active Motif, Carlsbad, CA, USA) following the manufacturer’s protocol.

Whole-cell protein extraction was done using RIPA buffer (Thermo Fisher Scientific Canada). The manufacturer’s protocol was followed with some modifications. Briefly, cells were washed with PBS prior to addition of trypsin to detach the cells. Cells were resuspended in complete medium and centrifuged. The cell pellet was rinsed with PBS and was resuspended in RIPA buffer with protease and phosphatase inhibitors. The sample was incubated on ice for 15 min, and centrifuged at 14,000*g* for 15 min. The supernatant was transferred into a pre-chilled micro-centrifuge tube for downstream application(s) or storage at −80 °C.

Protein extracts were run on Bolt™ 4-12% Bis-Tris Plus gels at 180V for 50 min at RT. Transfer onto nitrocellulose membrane was performed for 1 h at 250 mAmp on ice. Membranes were blocked in 5% BSA-PBST for 30 min at RT prior to incubation with the primary antibody at 4 °C overnight on a shaking platform. Following primary antibody incubation, membranes were washed three times with PBST (5 min/wash) and incubated with the HRP-conjugated secondary antibody for 1 h at RT on a shaking platform. Antibodies used are listed in Supplementary Table S1. To remove previous antibody probing(s), membranes were stripped using 1× stripping buffer (Millipore Sigma Canada). The ECL detection reagent kit (Amersham (under Cytiva), Mississauga, ON, Canada) was used to visualize the bands via the Bio-Rad VersaDoc Imaging System or the Bio-Rad ChemiDoc™ Touch. Raw membrane images were analyzed using Bio-Rad Image Lab 6.0.1 (software v 2.0.0.25).

### 2.9. Proximity Ligation Assay (PLA)

PLA experiments were performed according to the manufacturer`s guidelines (Sigma-Aldrich Canada) when using our own blocking reagent (5% BSA-PBST) and antibody diluent (1% BSA-PBST). A hydrophobic pen was used to mark the areas surrounding the coverslips for the PLA experiments. Briefly, PFA-fixed cells were incubated in 5% BSA-PBST for 30 min at RT for blocking and permeabilization. Samples were then incubated with the primary antibodies—1:100 mouse anti lamin A/C (sc376248) and 1:100 rabbit anti phos-PKC-α (sc12356-R) at RT for 1.5 h. The negative control was incubated in phos-PKC-α antibody only. After washing in PBST three times (5 min/wash), the manufacturer’s protocol was followed from here onwards, beginning with the incubation of the samples with the PLA probes. Z-stack images of samples were taken at 60× (1.40 NA oil objective) using the Nikon A1RsiMP Confocal Workstation (NIS-Elements AR 4.3 software). Channel series was activated to prevent spectral bleed-through between the channels. The 405 and 561 nm lasers were used. Quantitative analysis of PLA results was manually performed using ImageJ 1.47v 2.0.0-rc-43/1.52n (for Windows 64-bit) according to [65] except that a z-projection of the z-stacks for each nucleus was analyzed instead of a single stack per nucleus.

### 2.10. Data Analysis

GraphPad Prism 8.3.0 (San Diego, CA, USA) was used to perform statistical analyses. One-way ANOVA with Dunnett’s correction for multiple tests or two-tailed t-test (with or without Welch’s correction) was performed for analyses. Significance was accepted at *p* < 0.05.

## 3. Results

### 3.1. PKC-α Localization is Disturbed in Cells Expressing Various Striated Muscle Laminopathy Mutations

**Figure 1:**
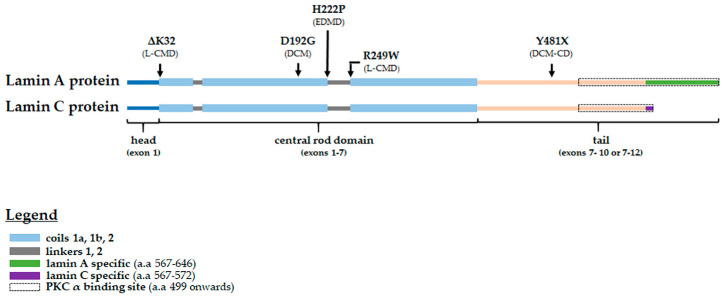
Lamin A/C protein schematic showing the locations of the pathogenic amino acid changes studied, their corresponding phenotypes and the PKC-α binding site (indicated by the black dotted line box). DCM—dilated cardiomyopathy; EDMD—Emery–Dreifuss muscular dystrophy; L-CMD—*LMNA* related congenital muscular dystrophy; and DCM-CD—dilated cardiomyopathy with conduction defects.

The locations of the pathogenic amino acid substitutions or deletions investigated in this study are shown in Figure 1. The amino acid changes span the entire protein, including locations in the head, central rod domain and tail. The phenotype (s) associated with each variant is shown in Table 1, including DCM, DCM-CD, EDMD and L-CMD.

**Figure 2:**
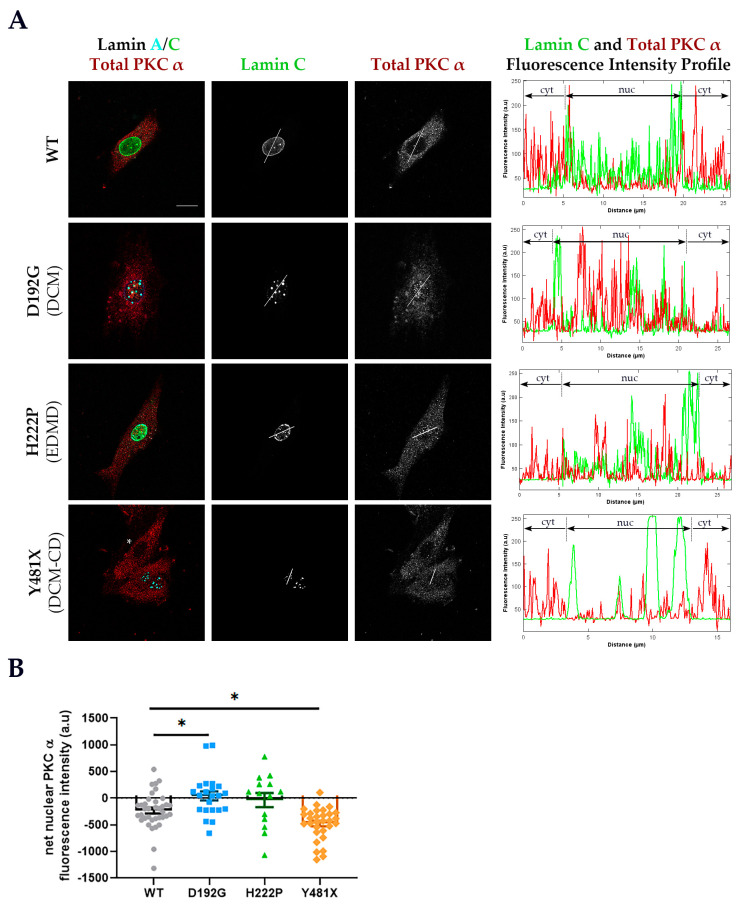
Cellular localization of total PKC-α in transfected H9C2 cells expressing either WT or mutant human lamins A and C. (**A**) Exogenous eCFP-human lamin A and eYFP-human lamin C are shown with the immunostaining for endogenous PKC-α (red) in WT and mutant *LMNA* expressing H9C2 cells. The composite image showing total PKC-α, eCFP-human lamin A and eYFP-human lamin C for each group is shown in the leftmost panel. A representative untransfected cell (indicated by a white asterisk) shows cytoplasmic PKC-α localization comparable to PKC-α localization in WT *LMNA* transfected cells. The white line across a cell in the lamin C and total PKC-α panels indicates the measurement path used to generate the fluorescence intensity profiles for lamin C and PKC-α shown in the rightmost panel. Since lamins A and C colocalize, the plot profile for eYFP-human lamin C only is included to show nuclear demarcation of fluorescence signal. Scale bar: 15 μm for all immunofluorescence images. (**B**) Mean (±SEM) net nuclear PKC-α fluorescence intensity of each group: WT (grey circles), D192G (blue diamonds), H222P (green triangle) and Y481X (orange diamonds)**.** The net nuclear PKC-α fluorescence intensity of each cell = adjusted nuclear PKC-α fluorescence intensity − mean of the adjusted cytoplasmic PKC-α fluorescence intensities. *n* ≥ 3 independent sets were analyzed (≥14 cells/group are analyzed). Significance (*) was set at *p* < 0.05.

To determine the effects of striated muscle laminopathy mutations on PKC-α cellular distribution, H9C2 rat cardiac myoblasts and C2C12 mouse skeletal myoblasts were transfected with fluorescently conjugated wild type (WT) human lamins A and C or fluorescently conjugated mutant human lamins A and C. The fluorescent marker allowed for identification of cells expressing human lamins A and C. Transfected H9C2 (Figure 2A) and C2C12 (Figure S1A) cells were immunostained for endogenous total PKC-α (both unphosphorylated and phosphorylated), and nuclear and cytoplasmic endogenous PKC-α fluorescence intensities were measured. From the nuclear PKC-α fluorescence intensity measurement, adjusted and net nuclear PKC-α fluorescence intensities were then calculated, and the net nuclear PKC-α fluorescence intensity was compared between the groups (Figure 2B, Figure S1B). PKC-α was primarily found in the cytoplasms (Figure 2 and Figure S1) of H9C2 and C2C12 untransfected cells and transfected cells expressing wild type lamins. In contrast, transfected cells expressing the p.D192G lamin A/C variant displayed a significant increase in nuclear PKC-α compared to WT (Figure 2 and Figure S1). On the other hand, transfected cells expressing the p.H222P lamin A/C variant showed PKC-α cellular localization that was comparable to WT (Figure 2). Likewise, the truncated p.Y481X lamin A/C that cannot bind PKC-α had PKC-α mostly in the cytoplasm (Figure 2) [21].

**Figure 3:**
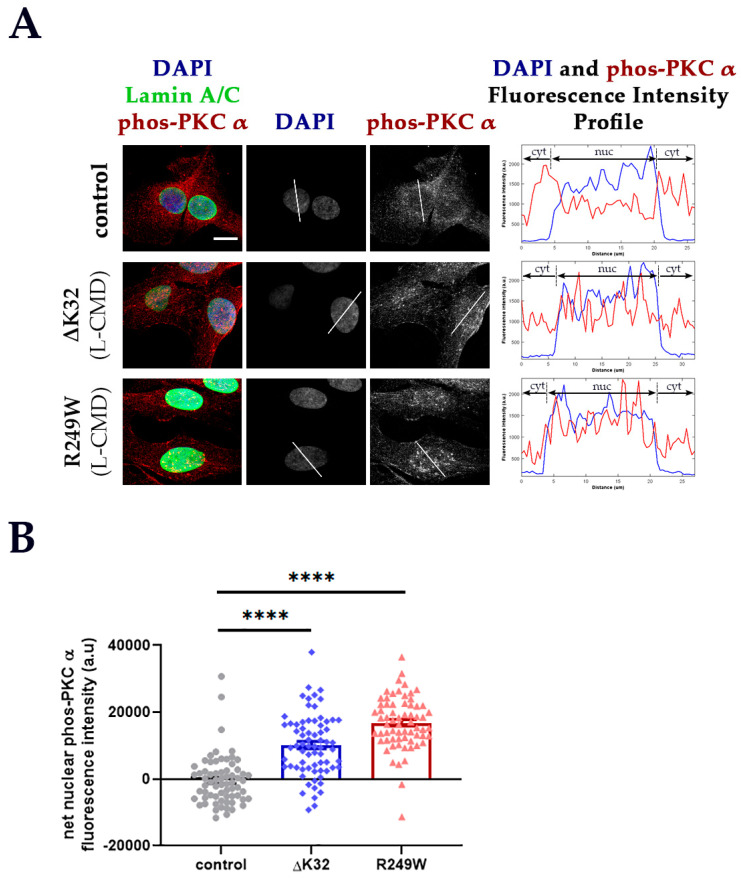
Cellular localization of phosphorylated PKC-α (phos-PKC-α) in control and patient myoblasts. (**A**) Immunostaining for DAPI, lamin A/C and phos-PKC-α in control and patient myoblasts. The composite image showing DAPI, lamin A/C and phos-PKC-α for each group is shown in the leftmost panel. The white line across a cell in the DAPI and phos-PKC-α panels indicates the measurement path used to generate the fluorescence intensity profiles for DAPI and phos-PKC-α shown in the rightmost panel. The plot profile for DAPI is included to show nuclear demarcation of fluorescence signal. Scale bar: 15 μm for all immunofluorescence images. (**B**) Mean (±SEM) net nuclear phos-PKC-α fluorescence intensity of each group: control (grey circles), ΔK32 (blue diamonds) and R249W (pink triangles)**.** The net nuclear phos-PKC-α fluorescence intensity of each cell = adjusted nuclear phos-PKC-α fluorescence intensity − mean of the adjusted cytoplasmic phos-PKC-α fluorescence intensities. *n* = 2 technical replicates (one patient/group; ≥ 66 cells/group were analyzed). Significance **** means *p* < 0.0001.

To confirm the results from the transfected cell lines, the cellular distribution of PKC-α phosphorylated at Ser657 (phos-PKC-α) was examined in human and mice myoblasts carrying lamin A/C mutations. Phosphorylated PKC-α was further studied, as this is the active form of the protein. Control and L-CMD patient myoblasts (one patient/variant) and myoblasts from WT mice, the EDMD H222P mouse model and the L-CMD p.K32del (ΔK32) mouse model were immunostained for endogenous phos-PKC-α. Phos-PKC-α was predominantly cytoplasmic in both the control human and mouse model myoblasts (Figure 3 and Figure S2). Similarly, phos-PKC-α’s cellular distribution in the H222P mice myoblasts remained mostly cytoplasmic (Figure S2). In contrast, both ΔK32 patient and mouse myoblasts displayed an increase in nuclear phos-PKC-α localization compared to control (Figure 3 and Figure S2).

To eliminate a factor that could have influenced PKC-α localization, *LMNA* over-expression was examined. Western blot analyses of nuclear extracts from sorted C2C12 cells expressing WT or mutant *LMNA* showed comparable levels of lamin A/C over-expression (Figure S3), suggesting that lamin A/C over-expression was unlikely to have caused the aberrant PKC-α localization.

To verify that the observed increase in PKC-α nuclear localization was not caused by a compromised nuclear membrane through which proteins could easily get into the nucleus, immunostaining for another protein—HSP60—was performed on human myoblasts (Figure S4). HSP60 is a chaperone protein found in various subcellular localizations, such as the cytoplasm, the mitochondria and the nucleus [70,71,72,73]. It has a molecular weight of 60 kDa, which is smaller than the 80 kDa PKC-α, thereby making it easier and more likely for HSP60 to get into the nucleus than PKC-α if the observed PKC-α mislocalization was due to compromised nuclear permeability. Qualitative analyses of patient myoblasts HSP60 immunostaining images showed that similarly to what was observed in the control myoblasts, HSP60 remained primarily outside of the nucleus in cells expressing mutant *LMNA* (Figure S4). This suggested that the increased nuclear localization of phos-PKC-α in the patient myoblasts was unlikely caused by a compromised nuclear membrane through which proteins could easily get into the nucleus. Overall, the results revealed PKC-α mislocalization in the presence of pathogenic *LMNA* mutations.

### 3.2. PKC-α Activation Is Disturbed by Lamin A/C Mutations

**Figure 4:**
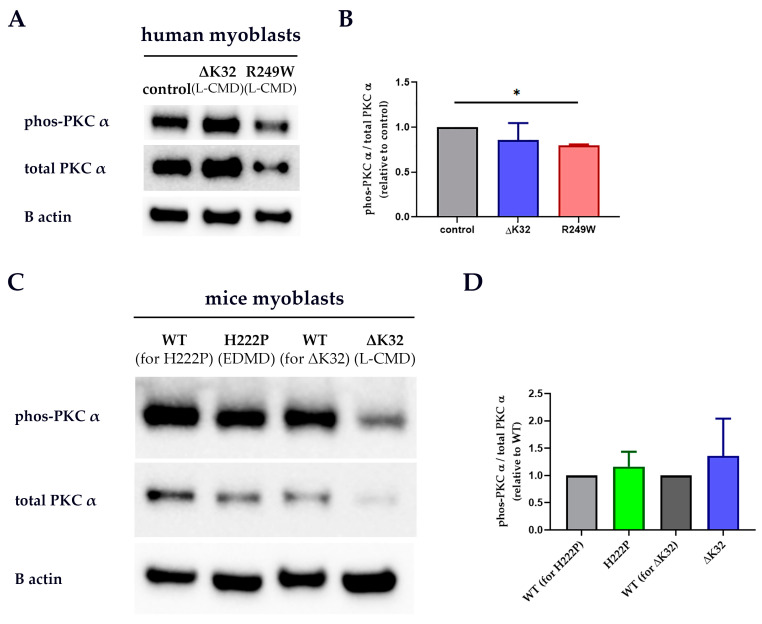
Western blot analyses for PKC-α in whole cell protein extracts from patient and mouse model myoblasts. Representative blots for human myoblasts (**A**) and mouse model myoblasts (**C**). Mean (± SEM) protein level relative to control (or WT) is shown for human myoblasts **(B)** and mouse model myoblasts (**D**). *n* ≥ 3 technical replicates (one patient or mouse model/group). Significance (*) was set at *p* < 0.05.

To determine whether the activation level of PKC-α was changed in the presence of pathogenic lamin A/C mutations, Western blot analyses of whole-cell protein extracts from human myoblasts and fibroblasts, and mouse model myoblasts were performed. PKC-α activation was significantly lower in the R249W patient myoblasts and fibroblasts compared to control (Figure 4A,B and Figure S5). In ΔK32 patient myoblasts and fibroblasts, a trend of reduced proportion of phos-PKC-α was observed (Figure 4A,B and Figure S5). On the other hand, while total PKC-α was significantly reduced in the H222P mouse model myoblasts compared to WT, neither the total PKC-α for the ∆K32 mouse model myoblasts nor the phos-PKC-α for both H222P and ∆K32 mouse model myoblasts were significantly different from their respective WT (data not shown); hence, the overall PKC-α activation level was comparable between the WT and the H222P and ΔK32 mice myoblasts (Figure 4C,D).

### 3.3. PKC-α and Lamin A/C Proximity Is Disrupted by Lamin A/C Mutations

**Figure 5:**
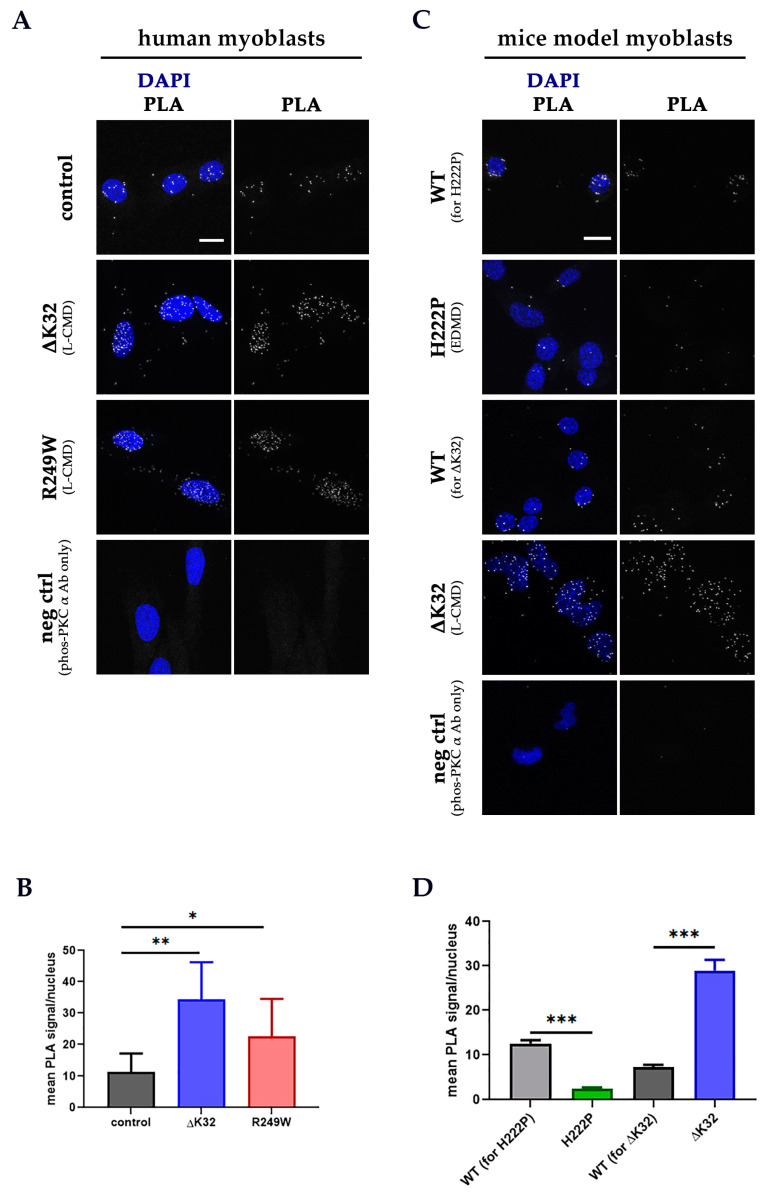
Proximity ligation assay (PLA) between lamin A/C and phos-PKC-α in human (**A**) and mouse model (**C**) myoblasts. Mean (±SEM) PLA signal/nucleus in human (**B**) and mouse model (**D**) myoblasts. Each nucleus was stained with DAPI (blue) and the PLA signal representing the proximity of lamin A/C and phos-PKC-α is in white. *n* = 2 technical replicates (one patient/group; ≥92 nuclei/group were analyzed) for human myoblasts and *n* = 1 experimental set (≥46 nuclei/group were analyzed) for mouse models’ myoblasts. Scale bar: 15 μm for all PLA images. Significance (*) was set at *p* < 0.05; ** means *p* < 0.01 and *** means *p* < 0.001.

To determine if *LMNA* mutations affected the proximity of PKC-α and lamin A/C to each other, proximity ligation assay (PLA) was performed on human myoblasts and fibroblasts, and mouse model myoblasts. A PLA signal is obtained if the target proteins are within 40 nm of each other [74]. Both ΔK32 and R249W human myoblasts and fibroblasts showed a significant increase in the number of PLA signals/nucleus compared to control (Figure 5A,B and Figure S6). Similarly, an increase in PLA signal was noted in the ΔK32 mice myoblasts, suggesting increased occurrence of lamin A/C and phos-PKC-α proximity in these cells (Figure 5C,D). On the other hand, the H222P mouse model myoblasts had fewer PLA signals than WT myoblasts (Figure 5C,D).

### 3.4. ERK 1/2 Activation Is Downregulated in Patient and Mice Model Myoblasts

**Figure 6:**
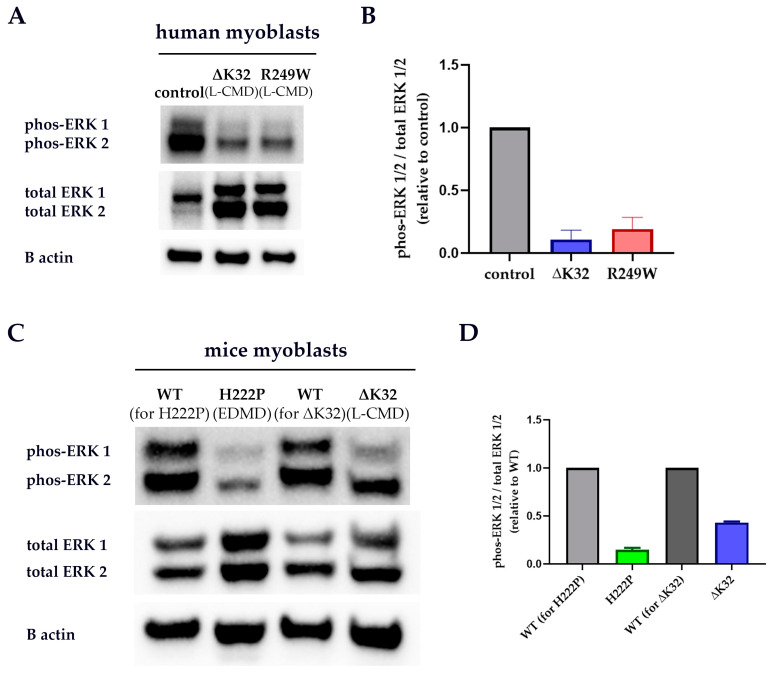
Western blot analyses for ERK 1/2 in whole cell protein extracts from patient and mouse model myoblasts. Representative blots for human myoblasts (**A**) and mouse model myoblasts (**C**). Mean (±SEM) protein level relative to control (or WT) is shown for human myoblasts (**B**) and mouse model myoblasts (**D**). *n* ≥ 2 technical replicates (one patient or mouse model/group).

ERK 1/2 is known to be regulated by PKC-α and is implicated in the development and progression of striated muscle laminopathies [44,45,46,50,51,52,53,56]. Western blots were performed to estimate phos-ERK 1/2 level in whole-cell protein extracts from human myoblasts and fibroblasts, and mouse model myoblasts. Phos-ERK 1/2 was drastically downregulated in both the ΔK32 and R249W patient myoblasts compared to control (Figure 6A,B). Likewise, ERK 1/2 activation was dampened in both the H222P and ΔK32 mice myoblasts (Figure 6C,D). However, phos-ERK 1/2 levels were similar in control and patient fibroblasts (Figure S5).

## 4. Discussion

Striated muscle laminopathies affect the majority of individuals carrying pathogenic lamin A/C mutations [2]. Unfortunately, there is currently no available cure or effective treatment for these patients. To further understand the molecular mechanisms contributing to the development and progression of striated muscle laminopathies, we studied PKC-α, one of lamin A/C’s kinase binding partners. Due to the central role of PKC-α in cardiac function, its interaction with the A-type lamins and its regulation of other lamin A/C partners, including ones implicated in striated muscle laminopathies, we sought to determine whether PKC-α is involved in cardiac and muscular diseases caused by lamin A/C mutations. Our study provides evidence linking PKC-α to striated muscle laminopathies by assessing PKC-α cellular localization, activation and proximity with the A-type lamins. We showed that PKC-α nuclear distribution was increased in most of the lamin A/C mutations that we studied. PKC-α is highly localized in the nuclei of cardiac and skeletal myoblast cell lines expressing a DCM-causing lamin A/C mutation. This increase in nuclear PKC-α localization was unlikely caused by lamin A/C over-expression or compromised nuclear permeability. Additionally, it was unlikely to have been caused by the fluorescent tag, as the L-CMD patient and ΔK32 mouse model myoblasts presented comparable results to the cell lines. The increase in nuclear PKC-α was paralleled by an increase in lamin A/C-phos-PKC-α PLA signal, suggesting more frequent proximity conducive to interaction of lamin A/C and phos-PKC-α in patient fibroblasts and myoblasts, and in ΔK32 mouse model myoblasts. We also show that in L-CMD patients’ myoblasts and fibroblasts, whole-cell PKC-α phosphorylation status is reduced, suggesting decreased PKC-α activation. Activation of ERK 1/2, a downstream PKC-α target previously associated with striated muscle laminopathies, is decreased in L-CMD patient myoblasts and in H222P and ΔK32 mice myoblasts. On the other hand, cells expressing the DCM-CD p.Y481X truncated lamin A/C lacking the PKC-α binding region in the C-terminus show more cytoplasmic (i.e., less nuclear) PKC-α subcellular localization than WT. Previous work determined that lamin A binds PKC-α downstream of amino acid 499 [21]. Thus, our data provide evidence to warrant further study of PKC-α in the context of pathogenic *LMNA* mutations.

Various patient and heart failure animal model studies found contradicting results regarding PKC-α expression. In cardiac tissues from various heart failure animal models and patients with heart failure due to dilated cardiomyopathy (DCM) or ischemic cardiomyopathy, PKC-α expression is upregulated [30,34,37,38,75]. In contrast, a rabbit heart failure model has reduced PKC-α expression in failing ventricles [36]. Decreased *PRKCA* expression in the left ventricle has also been associated with adverse cardiac remodeling and increased DCM risk in patients [35]. In our study, the proportion of phosphorylated PKC-α was downregulated in L-CMD patient myoblasts and fibroblasts. ERK 1/2 activation was reduced as well. PKC-α activation has been shown to be upstream of the ERK 1/2 cascade mediating cardiac myocyte hypertrophy [45,57]. Adverse cardiac remodeling is a hallmark of DCM, with cardiolaminopathy patients having improper cardiac remodeling, and many present mildly DCM (systolic dysfunction without severe left ventricle enlargement) [4,76]. In our study, the p.D192G and p.Y481X lamin A/C patients had DCM; the p.D192G patient presented a milder left ventricle dilation compared to the p.Y481X patient [63,69]. Interestingly, p.D192G cells had increased nuclear PKC-α, while p.Y481X cells mostly had cytoplasmic PKC-α. Considering PKC-α’s involvement in transducing cellular signals, aberrant PKC-α activation and localization might contribute to the abnormal cellular response to physical stimuli in cells expressing mutant lamin A/C, resulting in the development of a pathogenic phenotype in mechanically-stressed tissues such as the heart. The impaired ability of PKC-α to localize in the nuclei of p.Y481X cells potentially exacerbates the mechano-transduction defect in these cells, as PKC-α is less able to reach its nuclear targets, resulting in more pronounced cardiac remodeling (i.e., more significant left ventricle dilation) in the p.Y481X patient compared to the p.D192G patient [63,69]. On the other hand, in myoblasts from one-month old H222P mice, PKC-α activation and cellular localization are comparable to WT. The H222P mice do not present any notable phenotype at 4 weeks old [50,60].

Regarding lamin A/C interaction with a binding partner, one of the ways by which lamin A/C regulates proteins is by sequestering the target protein and/or its regulator or substrate [43,77,78,79]. Protein interaction mediated by the A-type lamins have been documented, such as in the lamin A/C-c-Fos-ERK 1/2 complex [77]. In this example, lamin A/C anchors both ERK 1/2 and c-Fos, enabling ERK 1/2 to phosphorylate c-Fos and free it from lamin A/C. Upon release from the nuclear lamina, c-Fos is then able to dimerize with c-Jun and assemble the AP-1 transcription factor [77]. In the present study, the formation of the lamin A/C-ERK 1/2-PKC-α complex is plausible, as lamin A/C is able to bind both ERK 1/2 and PKC-α [21,77]. In cells expressing p.D192G, p.K32del or p.R249W lamin A/C, an increase in nuclear PKC-α is observed. Concomitant with this increase in nuclear PKC-α is an increase in lamin A/C-PKC-α proximity. This enhanced proximity between lamin A/C and PKC-α likely keeps PKC-α in the nucleus by tethering PKC-α to the A-type lamins. However, structural changes incurred due to the lamin A/C mutations could have disrupted lamin A/C-ERK 1/2 interaction, thereby impeding the ERK 1/2-PKC-α interaction and resulting in decreased ERK 1/2 activation. Recent work on CRISPR knock-in mutant C2C12 myoblasts expressing p.R249W also showed a lack of ERK 1/2 activation in Western blot analyses compared to control [80]. Disruption of lamin A/C–ERK 1/2 association is plausible, especially in the p.R249W variant, as amino acid 249 is located in the region that binds ERK 1/2 [77]. However, even if the affected amino acid is not located in the PKC-α or ERK 1/2 binding sites, such as in the p.K32del variant, previous work has noted disruption of the lamin A/C-partner interaction [18]. Studies have also shown misassembly of p.K32del filaments potentially disturbing lamin A/C partner binding and/or interactions mediated by lamin A/C [19,81]. Alternatively, the lamin A/C-ERK 1/2 interaction might not have been affected but the overall conformational change in the lamin A/C variants might have disrupted the ERK 1/2-PKC-α interaction, resulting in reduced ERK activation. Similarly, downregulation of ERK 1/2 activation was noted in H222P mouse myoblasts. However, in these myoblasts, a decrease in PKC-α-lamin A/C proximity was observed. The reduction in lamin A/C-PKC-α proximity possibly impeded the lamin A/C-mediated ERK 1/2-PKC-α interaction, resulting in downregulation of ERK 1/2 activation. Previous work by another group on H222P myoblasts also does not note ERK 1/2 activation at baseline [50]. In addition, C2C12 cells stably expressing p.H222P lamin A show elevated ERK 1/2 activation only after AICAR treatment, glucose starvation or osmotic shock [51,82]. ERK 1/2 hyperactivation is also noted in C2C12 and Cos7 cells transiently transfected with p.H222P lamin A and in striated muscle tissues from symptomatic H222P mice, suggesting the involvement of physiological stress in upregulating ERK 1/2 phosphorylation in the context of the p.H222P lamin A/C variant [50,51,82]. Since the myoblasts we studied were collected from one-month old H222P mice which were still asymptomatic, physiological stress might not have been significant enough to result in notable ERK 1/2 activation [50,60]. Furthermore, a corresponding increase in total ERK 1/2 (especially ERK 2) was noted with a decrease in phos-ERK 1/2 in the mutant cells. This makes sense, as when fewer ERK 1/2 are phosphorylated, unphosphorylated ERK 1/2 will accumulate and increase in level. Previous studies have shown that ERK 2 is more abundant than ERK 1 in mice and zebrafish tissues [83,84]. Moreover, it has been demonstrated that either ERK can compensate for the lack of the other, as noted in the *Erk 1* null H222P mice, which had improved cardiac function and survival, but eventual ERK 2 upregulation and compensation overrode the benefit of *Erk 1* deletion [55,83,85]. MEK inhibitor treatment of the *Erk 1* null H222P mice restored the benefit of *Erk 1* deletion in these mice [55]. Dysregulation of a lamin A/C partner’s function as a consequence of disrupted lamin A/C binding has been demonstrated with other lamin A/C mutations. For instance, several lamin A/C variants with amino acid substitutions affecting the region that binds the transcription factor SREBF-1 (important for adipogenesis and metabolism) show impaired SREBF-1 binding [18]. This reduced binding resulted in SREBF-1 dysregulation, and abnormal expression of downstream SREBF-1 target genes contributing to lipodystrophy in patients [16]. Likewise, the lamin A/C variants p.R527P and p.L530P which have amino acid substitutions affecting the region that binds actin present with notable decreases in actin binding [17]. Individuals expressing either of these lamin A/C variants have EDMD [10]. Actin is involved in the transduction of mechanical signal in the cell and is part of the network that connects the cytoskeleton to the nucleus. Therefore, disruption of its association with the A-type lamins unsurprisingly results in a phenotype involving the muscles, which are tissues under constant mechanical and physical stress.

In conclusion, our results show that PKC-α cellular localization, activation and proximity with the A-type lamins are affected by striated muscle laminopathy mutations. Furthermore, the data hint at a potential correlation between the severity of the patient phenotype and abnormal PKC-α cellular localization, activation and proximity with lamin A/C. Results from cells expressing p.H222P (EDMD) or p.Y481X (DCM-CD) lamin A/C suggest that a predominantly cytoplasmic PKC-α localization, in combination with decreased PKC-α-lamin A/C proximity and decreased PKC-α and ERK 1/2 phosphorylation, results in a less severe muscular and/or cardiac phenotype. On the other hand, cells expressing variant lamin A/C (e.g., p.D192G, p.K32del, p.R249W) with increased nuclear PKC-α localization, increased PKC-α-lamin A/C association and decreased PKC-α and ERK 1/2 activation result in more severe cardiac and/or skeletal laminopathy. Future studies evaluating the ERK 1/2-lamin A/C interaction in cells expressing mutant lamin A/C are needed to determine the effects of the mutations on lamin A/C-ERK 1/2 binding. In addition, future work to determine ERK 1/2-lamin A/C-PKC-α assembly, and to characterize the interplay between these proteins, can also be performed. Other downstream PKC-α targets can be examined as well to further understand the roles of PKC-α in striated muscle laminopathies, and to identify other proteins that could potentially be therapeutic targets to treat striated muscle laminopathies.

## Figures and Tables

**Table 1:** Phenotypes associated with the studied *LMNA* variants. DCM—dilated cardiomyopathy; DCM-CD—dilated cardiomyopathy with conduction defects; EDMD—Emery–Dreifuss muscular dystrophy; and L-CMD—*LMNA* related congenital muscular dystrophy.

Variant	Phenotype (s)	Reference (s)
p.delK32	L-CMD *, severe EDMD	[66,67]
p.D192G	severe DCM	[63,68]
p.H222P	EDMD with arrhythmia	[9]
p.R249W	L-CMD	[11]
p.Y481X	DCM-CD	[63,69]

* Patient in this study was diagnosed with L-CMD.

## Supplementary Materials

The following are available online at https://www.mdpi.com/2073-4409/9/11/2388/s1. Figure S1: Cellular localization of PKC-α in transfected C2C12 cells expressing either WT or mutant human lamins A and C. Figure S2: Cellular localization of phosphorylated PKC-α (phos-PKC-α) in WT and mouse model myoblasts. Figure S3: Exogenous (transfected) and endogenous lamin A/C protein level analyses of nuclear extracts from transfected cells and sorted C2C12 cells expressing WT or mutant human lamins A (fused to eCFP) and C (fused to eYFP). Figure S4: Immunostaining for HSP60 (in magenta) and DAPI (in blue) in control and patient myoblasts. Figure S5: Western blot analyses for PKC-α and ERK 1/2 levels in whole cell protein extracts from human fibroblasts. Figure S6: Proximal ligation assay (PLA) between lamin A/C and phos-PKC-α in human fibroblasts. Table S1: List of antibodies and DNA stain used in the study.
