# Supplementary material for: Protein Kinase C Alpha Cellular Distribution, Activity, and Proximity with Lamin A/C in Striated Muscle Laminopathies
Source: Cells. 2020 Oct 31;9(11):2388. doi: 10.3390/cells9112388 (PMC7693451; doi:10.3390/cells9112388)
Supplement: Supplementary file 1 [file cells-09-02388-s001.pdf]

**Supplementary Information**

**A**

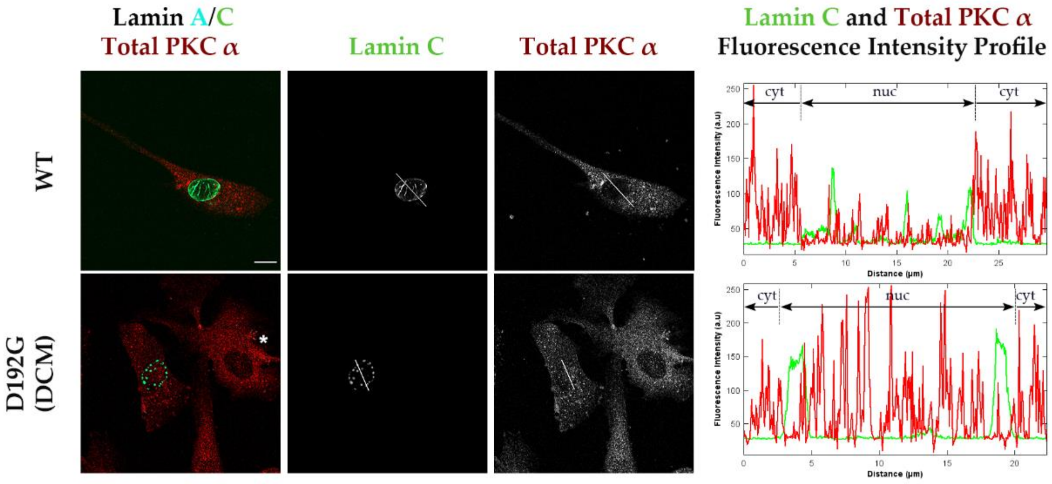


**B**

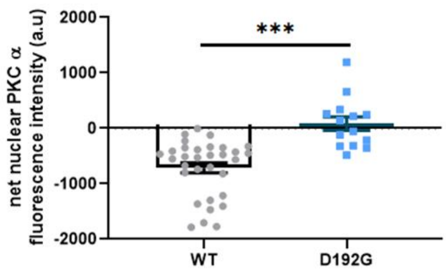


**Figure S1**: Cellular localization of PKC-$\alpha$ in transfected C2C12 cells expressing either WT or mutant human lamins A and C. **(A)** Exogenous eCFP-human lamin A and eYFP-human lamin C are shown with the immunostaining for endogenous PKC-$\alpha$ (in red) in WT and mutant *LMNA* expressing C2C12 cells. The composite image showing total PKC-$\alpha$, eCFP-human lamin A and eYFP-human lamin C for each group is shown in the leftmost panel. A representative untransfected cell (indicated by a white asterisk) showing cytoplasmic PKC-$\alpha$ localization comparable to PKC-$\alpha$ localization in WT *LMNA* transfected cells. The white line across a cell in the lamin C and total PKC $\alpha$ panels indicates the measurement path used to generate the fluorescence intensity profiles for lamin C and PKC-$\alpha$ shown in the right most panel. Since lamins A and C colocalize, the plot profile for eYFP-human lamin C only is included to show nuclear demarcation of fluorescence signal. Scale bar: 15 µm for all immunofluorescence images. (**B**) Mean (±SEM) net nuclear PKC-$\alpha$ fluorescence intensity of each group: WT (grey circles) and D192G (blue squares). The net nuclear PKC-$\alpha$ fluorescence intensity of each cell = adjusted nuclear PKC-$\alpha$ fluorescence intensity – mean of the adjusted cytoplasmic PKC-$\alpha$ fluorescence intensities. $n \geq 3$ independent sets (≥14 cells/group were analyzed). Significance (*) was set at $p < 0.05$ and *** means $p < 0.001$.

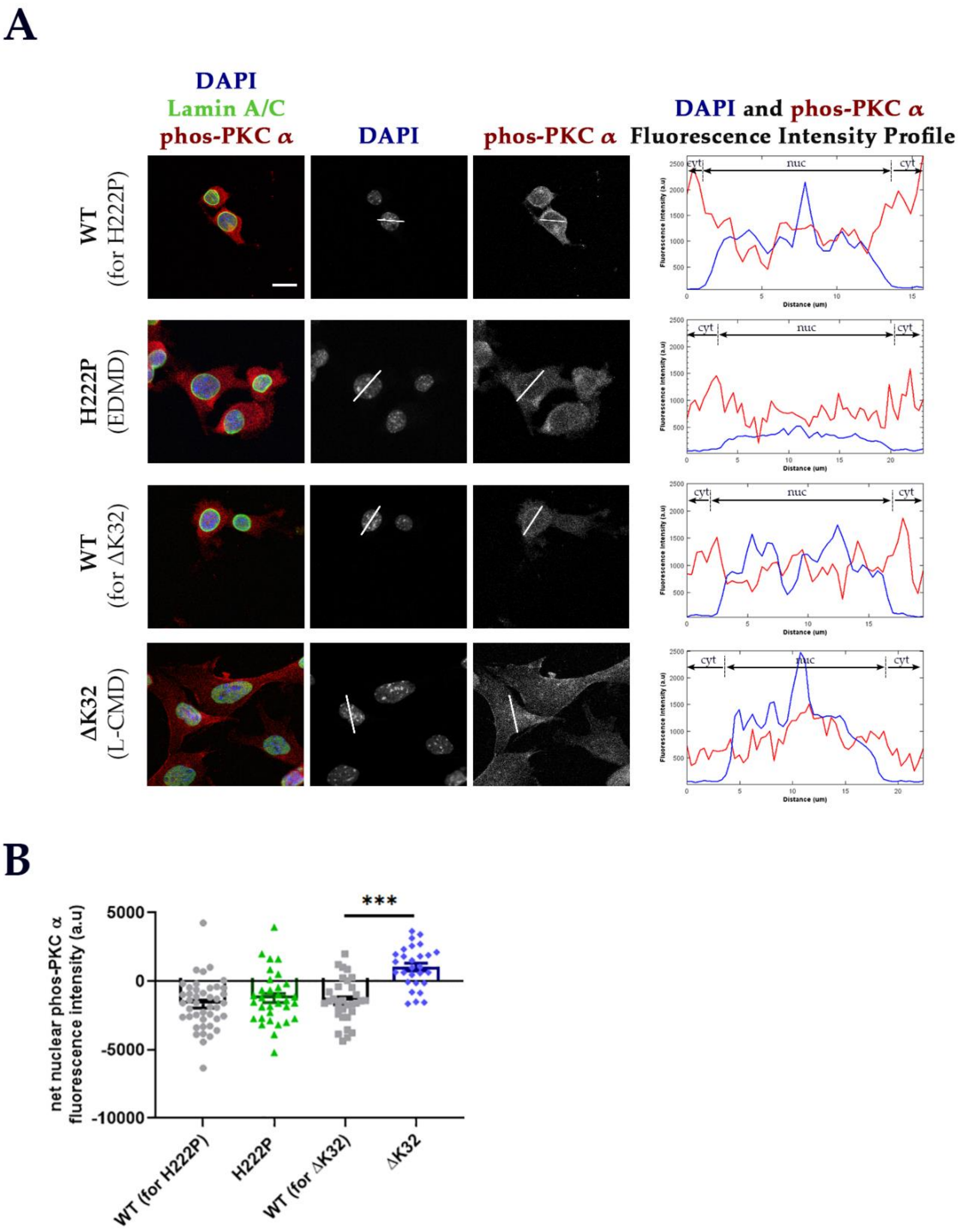


**Figure S2:** Cellular localization of phosphorylated PKC-$\alpha$ (phos- PKC-$\alpha$) in WT and mice model myoblasts. **(A)** Immunostaining for DAPI, lamin A/C and phos- PKC-$\alpha$ in WT and mice model myoblasts. The composite image showing DAPI, lamin A/C and phos- PKC-$\alpha$ for each group is shown in the leftmost panel. The white line across a cell in the DAPI and phos- PKC-$\alpha$ panels indicates the measurement path used to generate the fluorescence intensity profiles (right most panel) for DAPI and phos- PKC-$\alpha$. The plot profile for DAPI is included to show nuclear demarcation of fluorescence signal. Scale bar: 15 μm for all immunofluorescence images. **(B)** Mean (±SEM) net nuclear phos- PKC-$\alpha$ fluorescence intensity of each group: WT for H222P (grey circles), H222P (green triangles), WT for ΔK32 (grey square) and ΔK32 (blue diamonds)**.** The net nuclear phos- PKC-$\alpha$ fluorescence intensity

of each cell = adjusted nuclear phos- PKC-$\alpha$ fluorescence intensity – mean of the adjusted cytoplasmic phos- PKC-$\alpha$ fluorescence intensities. $n$ = 1 experimental set (≥30 nuclei/group were analyzed). Significance (*) was set at $p < 0.05$; *** means $p < 0.001$.

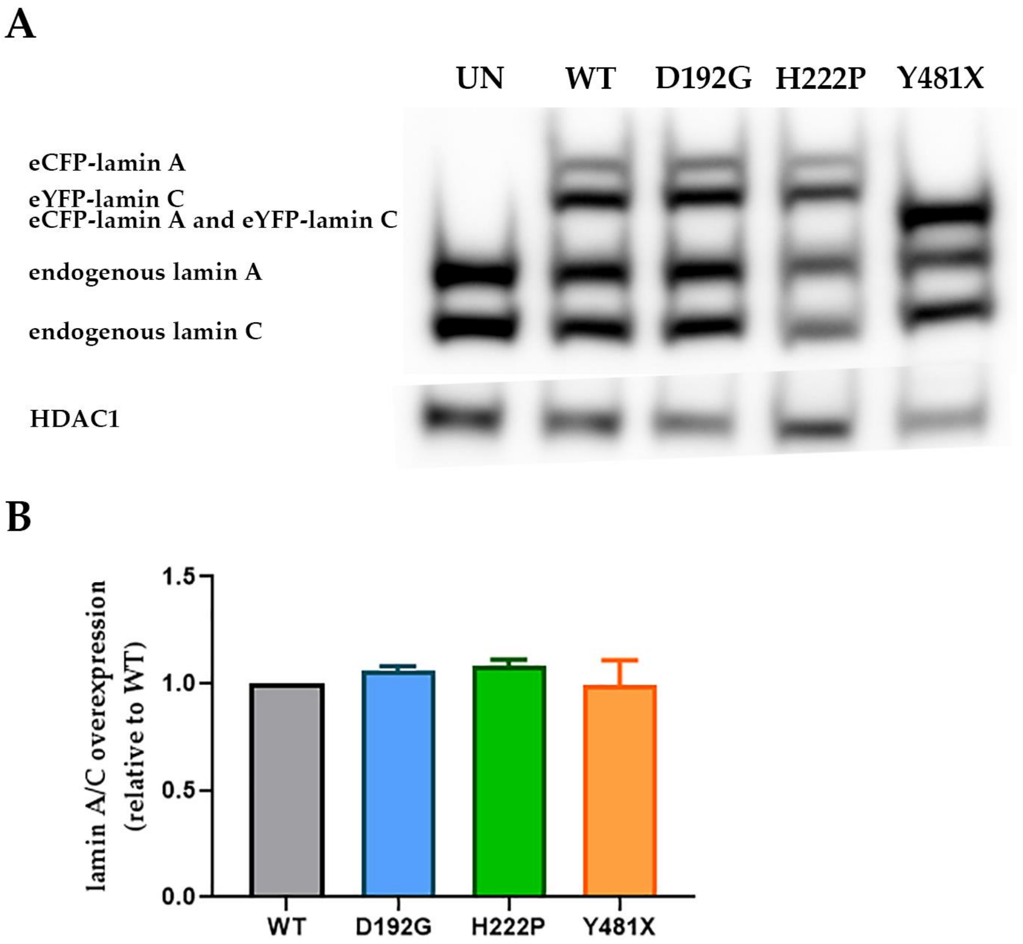


**Figure S3:** Exogenous (transfected) and endogenous lamin A/C protein levels analyses of nuclear extracts from transfected and sorted C2C12 cells expressing WT or mutant human eCFP-lamin A and eYFP-lamin C. **(A)** Representative western blot for lamin A/C in untransfected (UN) cells, WT and mutant *LMNA* expressing cells. **(B)** Comparison of lamin A/C overexpression between the WT and mutants. Mean ±SEM. $n \geq 2$ independent sets. Significance (*) was set at $p < 0.05$.

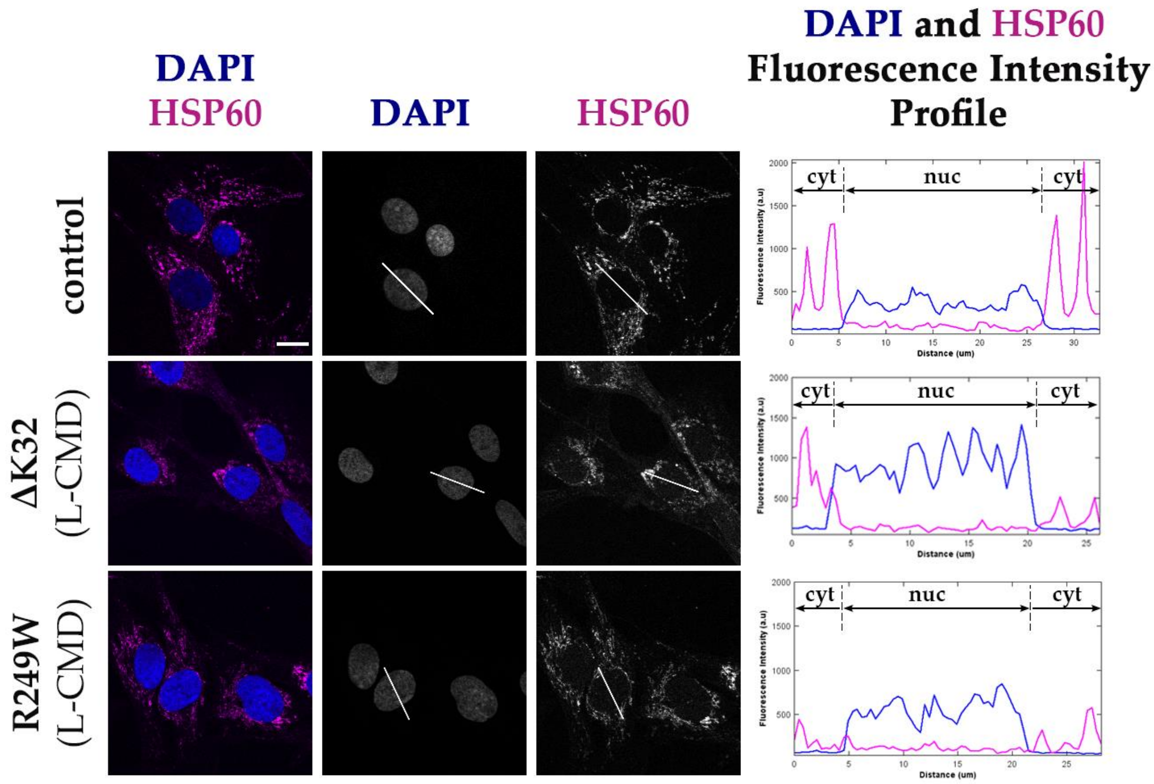


**Figure S4:** Immunostaining for HSP60 (in magenta) and DAPI (in blue) in control and patient myoblasts. The composite image showing DAPI and HSP60 for each group is shown in the leftmost panel. The white line across a cell in the DAPI and HSP60 panels indicates the measurement path used to generate the fluorescence intensity profiles for DAPI and HSP60 shown in the right most panel. The plot profile for DAPI is included to show nuclear demarcation of fluorescence signal. $n$ = 1 experimental set (one patient/group; ≥50 cells/group were analyzed). Scale bar: 15 μm for all immunofluorescence images.

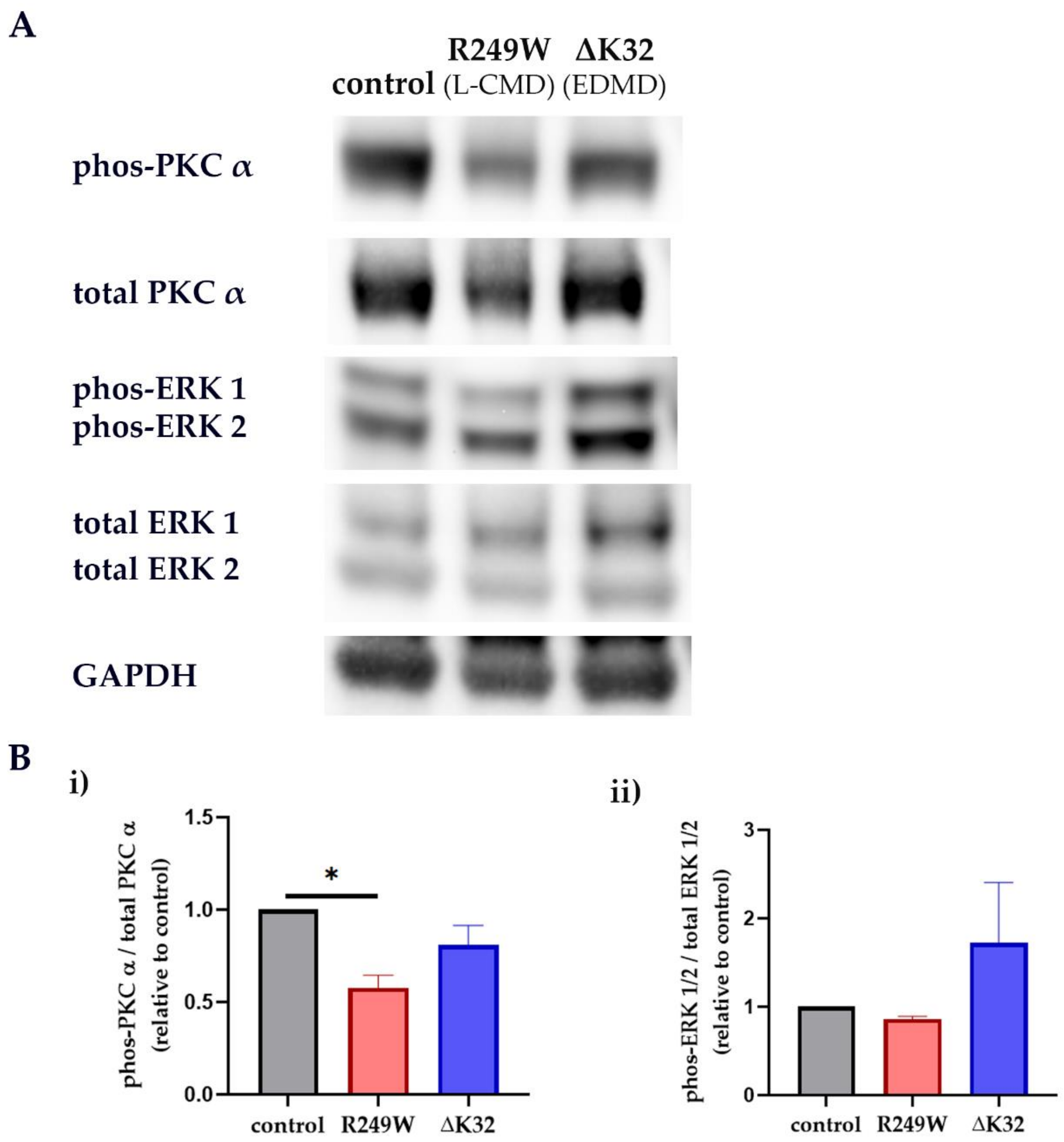


**Figure S5:** Western blot analyses for PKC-$\alpha$ and ERK 1/2 **l**evels in whole cell protein extracts from human fibroblasts. **(A)** Representative blots for PKC-$\alpha$ and ERK 1/2 probings. **(B)** Levels of activated PKC-$\alpha$ **(i)** and activated ERK 1/2 **(ii)** are graphed. Mean (±SEM) is shown. $n \geq 3$ technical replicates for PKC-$\alpha$ and $n \geq 2$ technical replicates for ERK 1/2 (one patient/group). Significance (*) was set at $p < 0.05$.

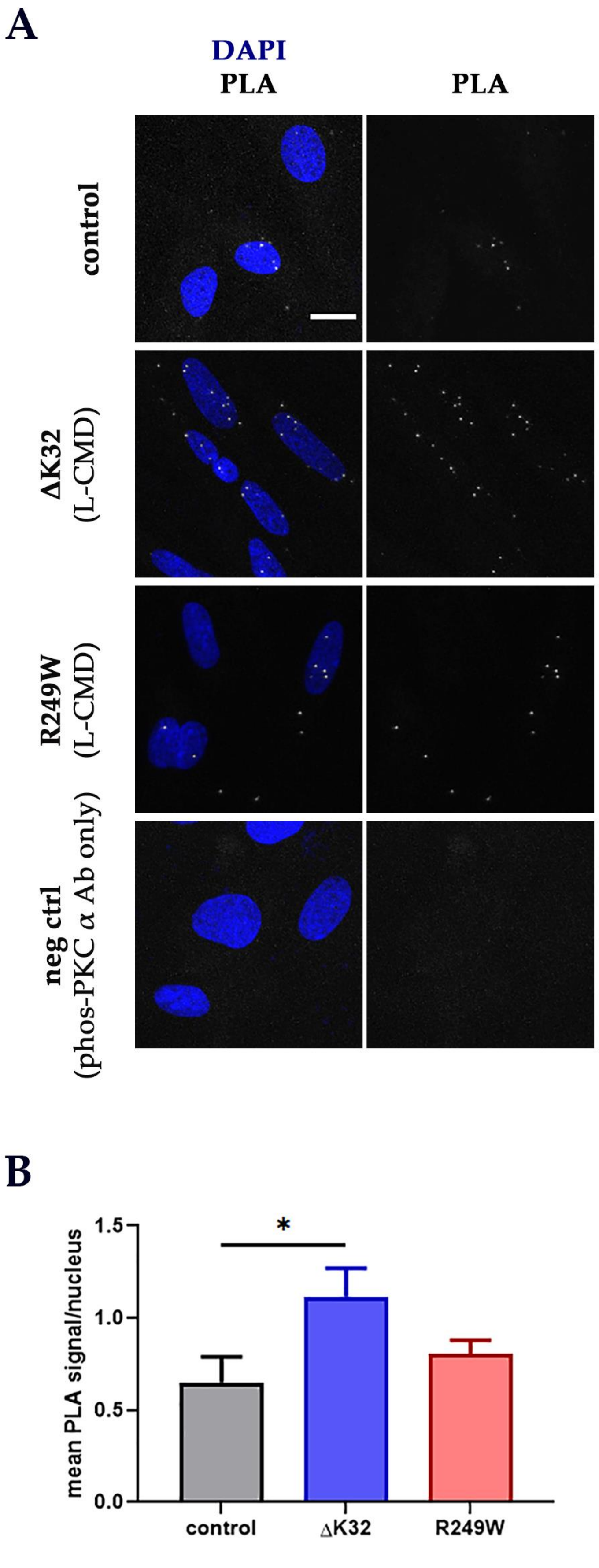


**Figure S6:** Proximity ligation assay (PLA) between lamin A/C and phos- PKC-$\alpha$ in human fibroblasts **(A)**. The nucleus is stained with DAPI (blue) and the PLA signal representing the proximity of lamin

A/C and phos- PKC-$\alpha$ is in white. Scale bar: 15 μm for all PLA images. **(B)** Mean (± SEM) PLA signal/nuclei in human fibroblasts**.** $n = 2$ technical replicates (one patient/group ≥115 cells/group were analyzed). Significance (*) was set at $p < 0.05$.

**Table S1.** List of antibodies and DNA stain used in the study. The respective dilutions for the application(s) are also indicated. IF means immunofluorescence, WB means western blot and PLA means proximity ligation assay.

| Antibody/Stain | Application(s) |
|---|---|
| mouse anti-total PKC-$\alpha$ (sc-8393) | IF (1:100), WB (1:500) |
| mouse anti-B actin (sc-47778) | WB (1:2000) |
| mouse anti-HSP60 (sc-13115) | IF (1:250) |
| mouse anti-lamin A/C (sc-376248) | IF/PLA (1:100) |
| rabbit anti-total PKC-$\alpha$ (sc-208) | IF (1:250), WB (1:2000) |
| rabbit anti-phos PKC-$\alpha$ (sc-12356-R) | IF/PLA (1:100), WB (1:2000) |
| rabbit anti-phos ERK 1/2 (Cell Signaling Technology 9101) | WB (1:2000) |
| rabbit anti-total ERK 1/2 (Cell Signaling Technology 9102) | WB (1:2000) |
| rabbit anti-HDAC1 (Ab33278) | WB (1:2000) |
| rabbit anti-GAPDH (Ab22555) | WB (1:15000) |
| goat anti-lamin A/C (sc-6215) | WB (1:2000) |
| anti-mouse Alexa Fluor 488 or 594 or 647 | IF (1:500) |
| anti-rabbit Alexa Fluor 488 or 594 | IF (1:500) |
| anti-rabbit or anti-mouse or anti-goat HRP-conjugated secondary | WB (1:5000) |
| DAPI | IF (1:5000) |
